# Design and discovery of metamorphic proteins

**DOI:** 10.1016/j.sbi.2022.102380

**Published:** 2022-05-10

**Authors:** Acacia F. Dishman, Brian F. Volkman

**Affiliations:** 1 Department of Biochemistry, Medical College of Wisconsin, Milwaukee, WI, USA; 2 Medical Scientist Training Program, Medical College of Wisconsin, Milwaukee, WI, USA

## Abstract

Metamorphic proteins are single amino acid sequences that reversibly interconvert between multiple, dramatically different native structures, often with distinct functions. Since the discovery of the first metamorphic proteins in the early 2000s, several additional metamorphic proteins have been identified, and it was suggested that up to 4% of proteins in the PDB may switch folds. Metamorphic proteins have been found to share common features such as marginal thermostability and inconsistencies in predicted secondary structures. Outstanding challenges in the field include the search for more metamorphic proteins and the design of new proteins that switch folds. Identification of novel metamorphic proteins in nature will improve therapeutic targeting of fold-switching proteins involved in human pathology and will enhance the design of protein-based therapies. Designed fold switching proteins have applications as biosensors, molecular switches, molecular machines, and self-assembling systems.

## Introduction

In the 70 years since Pauling, Corey and Branson predicted the α-helix and β-sheet, and the 50 years since Anfinsen’s thermodynamic hypothesis, our understanding of protein structure and folding coalesced around two main categories: globular proteins that find their native states spontaneously (either unaided or with help from chaperones) and fibrous proteins (multichain polymers typically assembled in the endoplasmic reticulum and the extracellular space). Then, in the late 1990s, intrinsically unfolded proteins and domains, which contain too few apolar sidechains to form a stable hydrophobic core, were recognized to comprise a significant fraction of the eukaryotic proteome [1].

**Figure 1:**
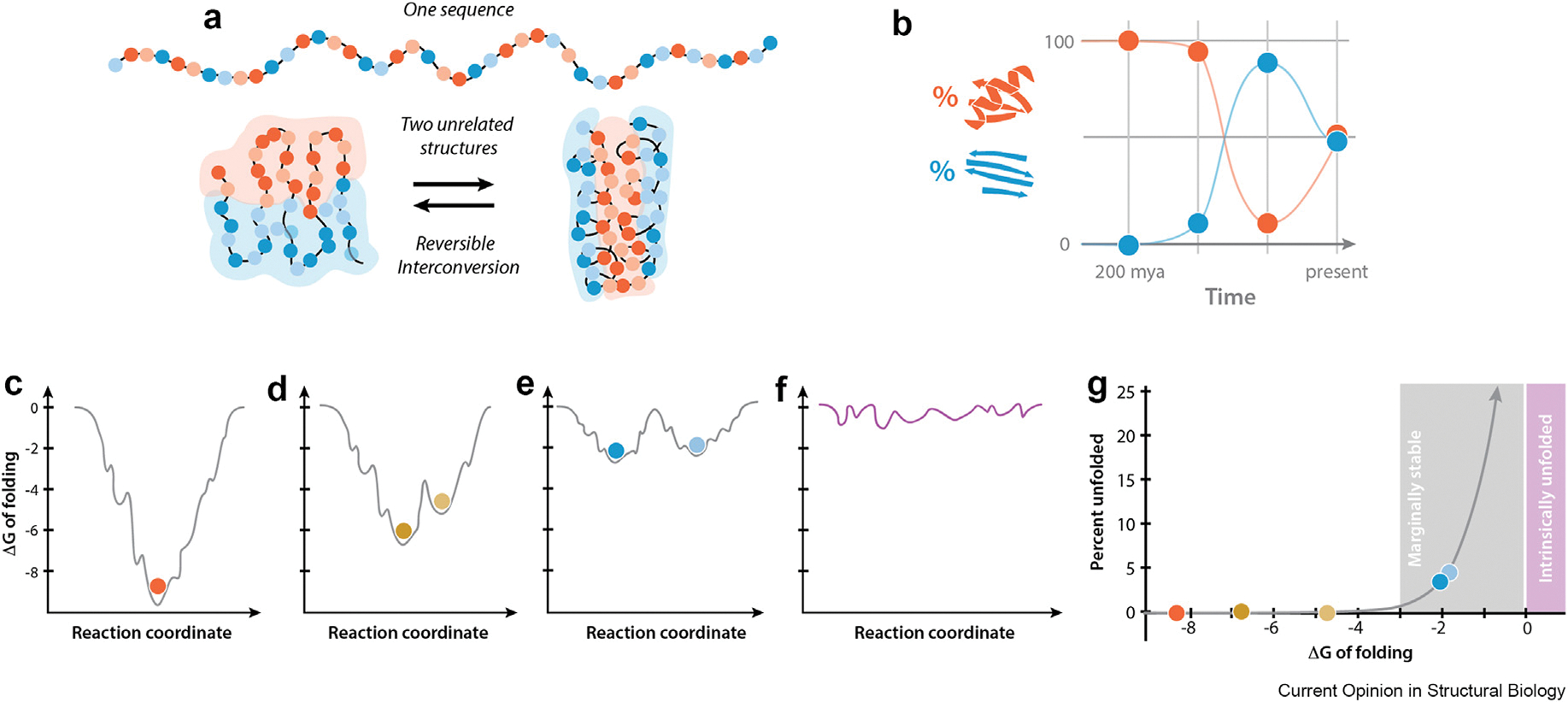
Thermodynamic characteristics of different categories of proteins. (**a**) Metamorphic proteins reversibly interconvert between two distinct, incompatible structures encoded by a single sequence. (**b**) Percent occupancy of the two XCL1 folds (chemokine fold, orange; β-sheet fold, blue) over evolutionary time, arriving at the modern day ratio of ∼50/50 (**c–f**) Hypothetical energy landscapes (i.e., folding funnels). for a stable monomorphic protein (**c**, a protein that undergoes subtle conformational change (**d**), a metamorphic protein (**e**), and an intrinsically unfolded protein (**f**). (**g**) Unfolded population versus ΔG of folding as calculated using ΔG_fold_ = −RT ln(K_eq_). Points from each energy landscape are plotted in their corresponding colors.

The 2002 discovery that XCL1 (previously known as lymphotactin) simultaneously adopts two unrelated native state structures again challenged the one sequence-one fold paradigm [2]. With the subsequent identification of several other examples, metamorphic proteins are now established as a distinct category with special properties and functions that remain largely unexplored (Figure 1a) [3]. Our most recent work used ancestral sequence reconstruction to trace the evolutionary history of XCL1 and identified the first ancestor that interconverted between two distinct conformations [4]. Dramatic shifts in the XCL1 metamorphic equilibrium from one ancestral node to the next suggest that the 1:1 ratio of β-sheet and chemokine conformations observed for human XCL1 is an optimized product of evolutionary selection (Figure 1b). Metamorphic folding was thus established as a favorable attribute that can improve protein fitness. In this article, we clarify the essential features and properties of a metamorphic protein, examine the search for more metamorphic proteins in nature, and assess the prospects for creation of metamorphic proteins as nano-scale tools or medical treatments.

## Observable characteristics of metamorphic proteins

Anfinsen’s thermodynamic hypothesis (the native structure is the conformation with the lowest free energy) and Levinthal’s paradox (rapid folding cannot be achieved via random search of possible configurations) launched the ‘protein folding problem’ and the search for stepwise folding pathways and intermediate states. As the database of solved structures grew, the native structure was widely presumed to be unique — that is, each protein sequence would encode only one lowest-energy configuration. However, as others have noted [5] this restrictive definition of the native state appears to be a misreading of Anfinsen. This concept is widely depicted as a ‘folding funnel’ which relates the number of conformational states (width) to the change in Gibbs free energy (depth) for the transition from unfolded (top) to native (bottom) conformation (ΔG of folding or ΔG_fold_) (Figure 1c).

Moreover, by the time Dill and Chan popularized the folding funnel concept in 1997 it was already clear that “*if landscapes are smooth, then native proteins should have small fluctuations, but if landscapes are rugged, then native proteins could fluctuate to very different conformations. Small changes in energy could lead to large changes in structure*” (Figure 1d,e). In theory, a polypeptide with two very different folded configurations of equal free energy would adopt both in equal proportions and fold-switching transitions would occur at a rate governed by the transition state energy. This would require them to have a folding funnel with multiple similarly shallow wells (Figure 1e) to permit interconversion via partial or complete unfolding, as was demonstrated for XCL1 [6]. Within a few years the first examples of proteins exhibiting these unusual features were described and eventually designated ‘metamorphic’ proteins by Alexey Murzin [3]. Intrinsically disordered proteins (IDPs) also fit naturally into this paradigm as featureless energy landscapes (Figure 1f).

Random sequence polypeptides do not fold spontaneously, and methods for computational design have only recently gained the ability to design stable structures with a deep folding funnel. If stable proteins are unusual, encoding more than one folded structure in a single amino acid sequence is a daunting prospect. Furthermore, facile switching between structures would require a particular type of conformational energy landscape ensuring that metamorphic proteins are relatively rare and exhibit specific biophysical characteristics (Box 1). Metamorphic proteins are more likely to exhibit marginal thermodynamic stability than singlefold proteins (Figure 1g) [7].

**Figure 2:**
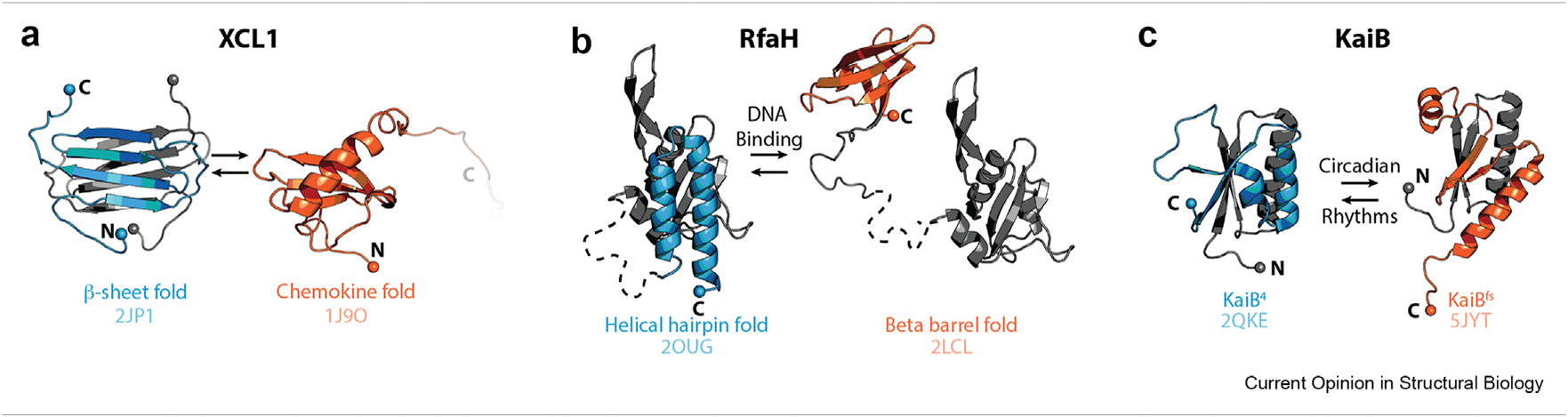
Metamorphic protein folding. (**a-c**) Example metamorphic proteins XCL1, RfaH, and KaiB. The dashed line in the RfaH structures represents a region which was not resolved in the RfaH crystal structure. N and C termini are labeled, and their alpha carbons are shown as spheres.

Because discerning the presence of multiple conformational states is a challenge for most biophysical methods, naturally occurring metamorphic proteins can go undetected and are probably underrepresented in the protein data bank (PDB). Metamorphic proteins switch between conformations at rates that prevent the chromatographic separation of each native structure and may be detrimental to crystallization. As nuclear magnetic resonance (NMR) spectroscopy is uniquely suited to the detection of conformational heterogeneity, this method has been widely employed in the characterization of metamorphic proteins, starting with XCL1 (Figure 2a) [2]. If the rate of interconversion between metamorphic structures is limited by partial or complete unfolding (i.e., slower than ∼10 s^−1^), a 2D heteronuclear single quantum coherence (HSQC) spectrum will contain distinct signals for each conformation with intensities proportional to their relative abundance in solution. Metamorphic behavior of well-studied metamorphic proteins RfaH (Figure 2b) and KaiB (Figure 2c) was also revealed by NMR analysis [8,9].

In a number of known metamorphic proteins, each fold encodes a distinct function [10,11]. For example, XCL1’s chemokine fold activates a G-protein couple receptor, while its β-sheet fold binds glycosaminoglycans (Figure 2a) [12]. One RfaH structure binds an *ops* DNA site, while the other structure binds a ribosomal subunit, meaning that fold-switching in RfaH couples bacterial transcription and translation (Figure 2b) [8]. The two folds of KaiB play different roles in a cyanobacterial circadian clock (Figure 2b) [13]. Other recent reviews catalogue known metamorphic proteins and their structure-function relationships in further detail [10,11,14,15]. Because identifying and separately characterizing the multiple conformations of metamorphic proteins is challenging, metamorphic protein functions may remain obscure long after the proteins are discovered. For example, XCL1 function was first described in 1994 [16], but it was not until 2008 that the two separate functions of XCL1’s two folds were disentangled [12]. Likewise, known proteins with single solved structures may have undetected alternate folds with alternate functions. Moonlighting proteins, which are known to have multiple functions [17], may thus be a particularly interesting group to search for previously undetected alternate folds.

## Searching for other natural metamorphic proteins

When Murzin coined the term “metamorphic protein” in 2008, he cited XCL1 and Mad2 as two prime examples. Since then, despite the popular notion that metamorphic proteins were likely exceedingly rare, several other metamorphic proteins have been serendipitously discovered [10,11,14,15], including SARS-CoV-2 protein ORF9b [18]. Experimentally determined structures describe 17% of the amino acid residues in human protein sequences [19]. Protein structure prediction has taken a quantum leap with AlphaFold [20], which was recently used to expand structural coverage, in the form of high confidence structure predictions, to 58% of all residues in the human proteome [21]. Different estimates suggest that between 15 and 30% of residues in the proteome are disordered [22,23]. This still leaves at least one tenth of all residues without a high confidence prediction *and* unlikely to be unstructured. Additionally, even proteins with high confidence structure predictions may have alternate folds that remain uncaptured.

A systematic search by Porter and Looger identified 96 fold-switching proteins in the PDB in 2018, and estimated that as much as 4% of proteins in the PDB switch folds [24]. Fold-switching proteins may be difficult to identify, however. In the traditional paradigm, one protein has one fold with one major function, and therefore once a single structure for a given protein is solved, additional structures are neither expected nor sought out. Additionally, metamorphic proteins are likely to be difficult to work with at the bench, particularly in the setting of structure determination by crystallography, due to their dynamic nature and low stability. Thus, metamorphic proteins are likely underrepresented in the PDB. It may also be difficult to generate high confidence structure predictions for metamorphic proteins due to their relative instability, as well as the fact that there may be a low-confidence prediction for each of their structures. Even predicting secondary structures for metamorphic proteins has been shown to generate inconsistencies [24–26].

Recently, these inconsistencies in secondary structure prediction have been harnessed to predict protein metamorphism. Of the 96 fold switching proteins identified by Porter and Looger, each represented by two structures, 85 pairs had at least one structure with substantial discrepancies between predicted and experimentally determined secondary structure [24]. Since then, secondary structure predictions for fold switching protein regions have been found to be consistently less accurate than secondary structure predictions for randomly selected, equally long regions of non-fold-switching proteins [26]. Alpha-beta discrepancies detected by secondary structure prediction algorithm JPred4 can also be used to predict whether protein families with similar sequences will have different folds, i.e., whether they will behave as “sequence-similar fold switchers” [25]. A recent preprint describes the use of such discrepancies in predicted secondary structure to identify fold-switching members of the >15,000-member NusG superfamily, which contains prototypical metamorphic protein RfaH [27]. The study predicted that ∼25% of the superfamily would switch folds. A sparse survey of 10 sequence-diverse variants by circular dichroism (CD) and NMR supported this prediction. Additionally, this study identified fold switching behaviors in variants with as little as 32% sequence identity, suggesting that diverse sequences can encode fold switching behavior, and supporting the idea that there may be more metamorphic proteins in nature than would otherwise be expected.

Identifying more fold-switching proteins in nature will allow fold-switching proteins to be therapeutically targeted. Fold switching can be involved in pathology, as in the case of the most common cancer-associated mutation (D83V) in the protein MEF2B, a major source of somatic mutations in non-Hodgkin lymphoma. This mutation causes an α-helical region to switch to a β-strand [28]. This indicates that a single amino acid change can trigger a fold switch implicated in cancer pathogenesis. Knowing how to identify fold-switching proteins, and sequence similar fold-switchers that switch folds in response to a few mutations, will enhance targeting of protein structure-function relationships in disease. Fold-switching can also be important for certain proteins’ physiologic roles in fighting disease. XCL1 switches between two dramatically different folds, one of which binds and activates its cognate GPCR on dendritic cell surfaces, and the other of which binds glycosaminoglycans and directly kills microbial pathogens via membrane disruption [12,29,30]. XCL1 variants locked in each of the two folds have been engineered by the Volkman lab [31,32]. Matsuo and coworkers sought to develop a cancer vaccine using XCL1 as an adjuvant but had limited success until they used an XCL1 variant locked in the GPCR-binding fold by an additional a disulfide bond [33]. In mice, at the injection site and draining lymph nodes, this locked variant then achieved the authors’ goal of inducing accumulation of dendritic cells capable of antigen cross-presentation to CD8+ T cells, triggering production of long-term antigen-specific memory CD8+ T cells [33]. This strategy would not have been possible without knowing that XCL1 has two folds with two functions. These examples highlight the importance of searching for more fold-switching proteins. If structural biologists stop after solving one structure for each protein, an entire universe of other protein folds may never be discovered.

## Design and engineering of fold switching proteins

**Figure 3:**
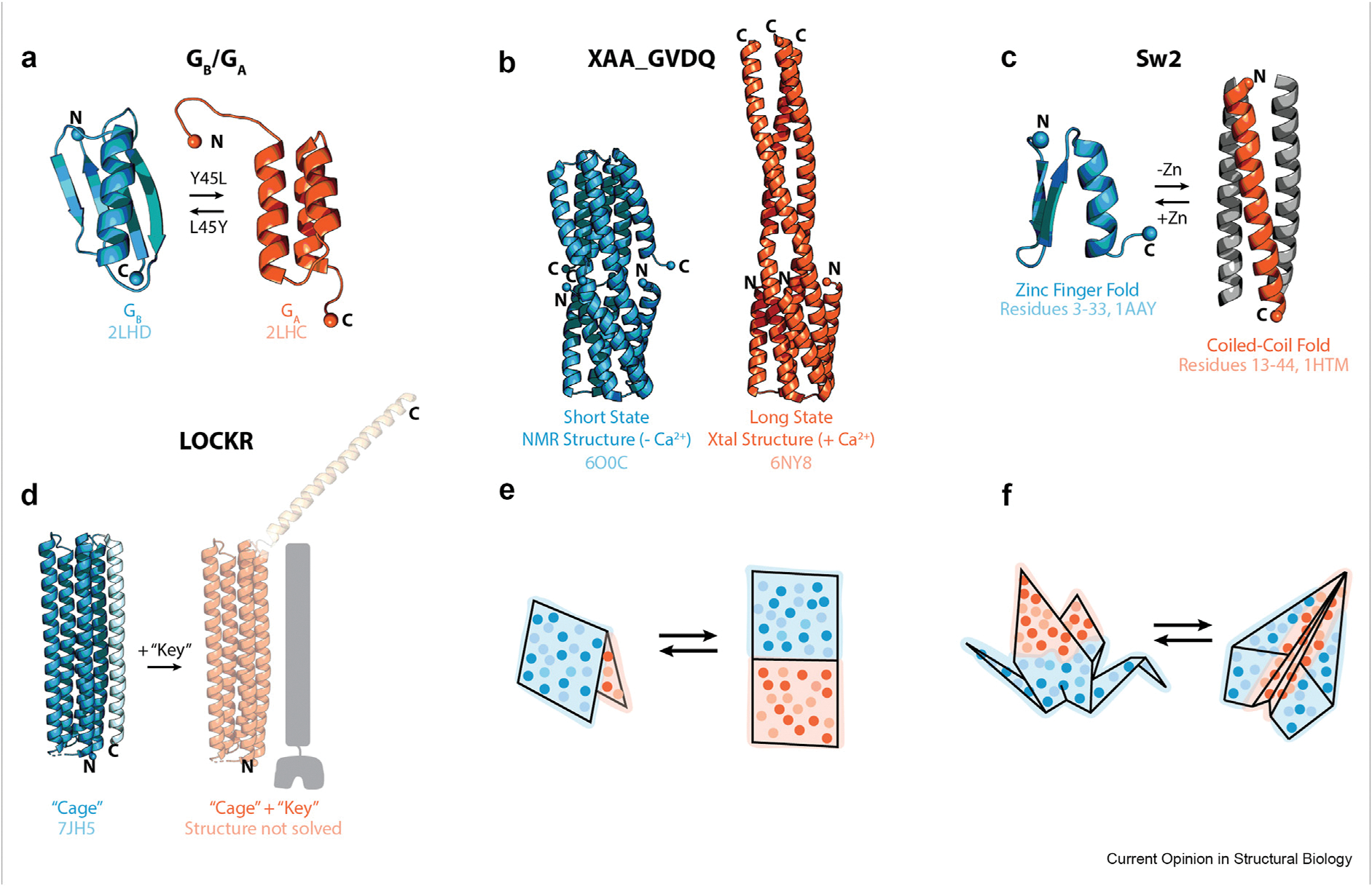
Design and applications of fold switching proteins. (**a–d**) Fold-switching proteins designed to date. The alpha carbons of the N and C termini are shown as spheres and labeled. PDB IDs are shown below each structure. (**a**) Proteins G_A_98 and G_B_98 differ by one amino acid and adopt different folds [37] (**b**) XAA_GVDQ is a *de novo* designed protein inspired by hemagglutinin which adopts one fold in a structure solved by NMR, and a significantly different fold in a structure solved by crystallography but has not been demonstrated to interconvert reversibly between the two structures [36]. (**c**) Sw2 is a computationally designed 30-residue protein that reversibly exchanges between a trimeric, coiled-coil, 3-α fold to a zinc finger fold in response to the addition of zinc [41]. (**d**) Co-LOCKR (Colocalization-dependent Latching Orthogonal Cage–Key pRotein) is a *de novo* designed protein system in which a “key” peptide displaces a “latch” helix from a 5-helix bundle [39]. The structure of the protein bound to the “key” peptide has not been solved. (**e**) *De novo* designed fold switching protein systems to date, such as Co-LOCKR, undergo hinge motions without substantially rearranging secondary structure or intramolecular contacts in the subunits that hinge with respect to one another. (**f**) Metamorphic proteins undergo large-scale conformational rearrangements often involving changes in secondary structure and substantial reorganization of intramolecular contacts.

Design of amino acid sequences not found in nature that (1) stably adopt *multiple*, *distinct* well-defined structures and (2) *reversibly* interconvert between them remains a challenge. To date, proteins have been designed that undergo subtle, designed structural fluctuations reversibly, such as proteins termed DANCERs in which a reporter tryptophan residue flips from a buried to a solvent exposed conformation [34]. Proteins have also been designed that adopt distinct structures but do not reversibly interchange between them, such as Hori and Suigura’s Ant-F [35] and Wei and colleague’s XAA_GVDQ (Figure 3b) [36]. Sequences which differ by one amino acid and adopt different folds have been designed as well (Figure 3a) [37]. Additionally, a recent Bryan lab preprint reports the design of identical amino acid sequences that adopt distinct structures based on the presence or absence of additional residues preceding or following the sequence [38].

The Baker lab has designed a biologically active protein switch system called co-LOCKR (Colocalization-dependent Latching Orthogonal Cage—Key pRotein) using Rosetta, which consists of two proteins [39,40] (Figure 3d). The first protein switches from a monomeric, helical bundle, closed or “latched” conformation to a heterodimeric, open state in response to the addition of the second protein, called the “key,” which binds at the same interface as the “latch” domain [39,40]. The switch in this system is not reversible and the open state is not structurally well defined [39,40].

In 2006, Ambroggio and Kuhlman designed fold-switching protein Sw2, marking the first use of computational protein design to optimize a single sequence for two different structures (Figure 3c) [41]. Notably, one of Sw2’s two folds had numerous exposed hydrophobic residues and thus aggregated unless suspended in buffer containing 200 mM guanidine. Similarly, Cerasoli and colleagues designed a 27-residue peptide that switches between two folds reversibly, using sequence comparison and visual pattern recognition rather than energy based calculations [42]. Since these examples, to our knowledge, a *de novo* designed protein that reversibly exchanges between two distinct structures has not been designed.

The challenge of consistently designing single sequences which interconvert reversibly between two distinct, arbitrary structures, particularly structures not found in nature, remains unsolved. Current protein design methods search for amino acid sequences that fold into a chosen structure with the lowest free energy, a strategy which has been successful in designing highly stable, monomorphic proteins, but poses challenges for the design of bistable proteins. Current protein design methods also struggle to predict folding energies of designed proteins at high accuracy, making it difficult to design a protein with two folds that have higly similar energies of folding, as is required for fold-switching. Even if two folds with similar energies are designed, the energy barrier between the two must not be so high as to prevent switching, which is currently difficult to predict using available protein design software. To circumvent this challenge, one can imagine designing proteins with two relatively unstable folds; however, the folds must then not be so unstable that the designed protein is intrinsically disordered or adopts a heterogenous mixture of conformations. In all, the design of bi-stable proteins is a formidable challenge.

New methods for bistable protein design, such as the Rosetta-based multistate design approach called RECON from the Meiler lab [43], show promise for designing sequences with multiple characteristics, e.g., multi-specific influenza antibodies [44]. Additionally, recent advances in structure prediction methodologies such as AlphaFold2 [20] will likely improve our ability to predict which designed proteins will be capable of stably adopting two different folds. Predictive methods such as a Baker lab deep learning approach have even been shown to be capable of detecting alternative minima in energy landscapes [45]. In a recent preprint, Jendrusch and colleagues embed AlphaFold in a novel protein design framework and use this framework to design sequences that are predicted to switch folds upon oligomerization with high confidence. While these designs remain untested in the laboratory, this framework demonstrates the way AlphaFold, or other structure prediction techniques, can advance design of fold switching proteins [46]. Together, these recent advances in multistate design and structure prediction provide hope for the future design of fold switching proteins.

*De novo* designed fold-switching proteins have diverse applications in healthcare and industry, and knowledge gained from the design process will have fundamental implications for understanding protein folding and dynamics [47]. For example, fold-switching proteins could be designed to act as molecular switches, for use as therapeutics, sensors, or components of cellular computers. Molecular switch systems have already been built using *de novo* designed proteins, such as the co-LOCKR system described above, or a system of *de novo* designed proteins which switch from monomer to trimer assemblies based on pH [39,40,48]. Co-LOCKR variants have been engineered to recognize certain combinations of surface antigens specific for cancer cells — e.g., Her2 and EGFR on the surface of breast cancer cells — switching “on” only in the presence of both antigens [49]. The goal is to use this system to target therapies specifically to cancer cells while sparing non-cancerous host cells [49].

Protein switch systems designed to date largely undergo hinge motions rather than the more substantial changes in intramolecular contacts and even secondary structure undergone by metamorphic proteins (Figure 3e,f). Metamorphic switches provide the opportunity for more complete rearrangement of the primary sequence in space, allowing multiple distinct spatial combinations of amino acids to be encoded in the same sequence. Moreover, metamorphic proteins that interconvert via complete or partial unfolding are likely to switch more slowly (e.g. seconds) than hinge-based (millisecond) conformational rearrangements. Slower switches would provide longer access to each fold-switched state, better enabling slower binding events. Each type of switch may be optimal for regulating different biological events. Control of molecular switch systems by fold switching proteins would provide an advantage over many current designs: reversible switching with the potential for tunable kinetics. Not only could fold switching proteins serve a unique role as components of molecular sensors, switches, and machines, but they could provide key information on the fundamental processes of protein folding and dynamics. Efforts to create fold switching proteins will test and refine the design principles for sequences that encode multiple 3D structures. Protein engineering successes should in turn facilitate the search for other fold switching proteins in nature.

## Box 1. What makes a protein metamorphic?

Proteins fold spontaneously into the lowest energy 3D configuration as originally shown by Anfinsen for RNase and illustrated by Wolynes as an energy landscape or ‘folding funnel.’ 3D structures solved (mainly by X-ray crystallography) through the 1970s, 80s and 90s cemented a particular interpretation of the thermodynamic hypothesis, namely that the amino sequence of a globular protein contains the information required to specify exactly one native state conformation, i.e., ‘one sequence, one fold’.

Nothing in Anfinsen’s description limits the native state of a polypeptide to a single structure. If the energy landscape contains two different energy minima of near-equal depth, each funnel represents a distinct 3D structure. If spontaneous folding results in both structures being populated and they are observed to interconvert, the protein may be metamorphic.

### Metamorphic proteins are single protein sequences that reversibly switch between multiple, dramatically different native structures, often with different functions.

What are the criteria or common characteristics for defining a metamorphic protein?

- **Marginal thermostability** (i.e., ΔG_fold_ > −3 kcal/mol), in contrast to ΔG_fold_ values ranging from −15 to −5 kcal/mol for most globular proteins.
- **Spontaneous unfolding.** Marginal thermostability implies that >1% of protein molecules are unfolded at any given moment (Figure 1g), making alternative native-state structures readily accessible.
- **Structural dissimilarity.** Helix–sheet conversions or changes to β-sheet topology will reorganize a large fraction of tertiary contacts within the metamorphic protein or independently folding domain. These changes can be catalogued and quantified using Protein Contact Atlas (https://www.mrc-lmb.cam.ac.uk/rajini) or other bioinformatic tools.

What is not a metamorphic protein? A partial list:

- **Chameleon sequences,** which are identical sequence fragments encompassed by larger sequences that differ
- **Prions,** which undergo irreversible fold changes
- **Local/subtle conformational changes,** which are less dramatic than the changes undergone by metamorphic proteins
- **Coupled binding/folding of IDPs,** which converts an unfolded protein to a folded protein, as opposed to converting one folded state to another
- **Irreversible PTM-driven conformational changes** (e.g., cleavage). However, certain PTM-induced changes may be viewed as metamorphic interconversion, particularly when a reversible modification (e.g., phosphorylation) could mimic a slow equilibrium process
- **Proteins that occupy different folds as a result of sequence modifications** such as mutations, extensions, or cleavage
